# Long Non-Coding RNA MEG3 Modifies Cell-Cycle, Migration, Invasion, and Proliferation Through AKAP12 by Sponging miR-29c in Meningioma Cells

**DOI:** 10.3389/fonc.2020.537763

**Published:** 2020-11-04

**Authors:** Chenyu Ding, Xuehan Yi, Jiaheng Xu, Zhenhua Huang, Xingyao Bu, Desheng Wang, Hongliang Ge, Gaoqi Zhang, Jianjun Gu, Dezhi Kang, Xiyue Wu

**Affiliations:** ^1^ Department of Neurosurgery, The First Affiliated Hospital of Fujian Medical University, Fuzhou, China; ^2^ Department of Otolaryngology Head and Neck Surgery, Fujian Medical University Union Hospital, Fuzhou, China; ^3^ Department of Neurosurgery, Zhengzhou University People’s Hospital, Henan Provincial People’s Hospital, Zhengzhou, China

**Keywords:** MEG3, miR-29c, AKAP12, meningioma, LncRNA

## Abstract

Meningioma (MEN) is a common central nervous system disease. Accumulating evidence indicated that long non-coding RNA maternally expressed gene 3 (MEG3) participated in the progression of MEN. However, the potential mechanisms of MEG3 in altering the aggressive phenotypes of MEN need further exploration. Levels of MEG3, microRNA (miR)-29c, and A-kinase anchor protein 12 (AKAP12) were determined using quantitative real-time Polymerase Chain Reaction (qRT-PCR) assay. Dual-luciferase reporter and RNA immunoprecipitation (RIP) assays were performed to verify the relationship between miR-29c and MEG3 or AKAP12. The protein level of AKAP12 was detected by western blot. Moreover, cell-cycle arrest, migration, invasion, and proliferation were assessed by flow cytometry, wound healing, transwell assays, and CCK-8 assay, respectively. Levels of MEG3 and AKAP12 were downregulated, while miR-29c was effectively increased in MEN tissues and cell line. Mechanically, MEG3 was a sponge of miR-29c to regulate the expression of AKAP12. Functionally, increase of MEG3 diminished cell-cycle, migration, invasion, and proliferation in MEN cells, and reintroduction of miR-29c could eliminate these effects. In addition, AKAP12 depletion overturned the inhibitory effects of miR-29c absence on cell-cycle, migration, invasion, and proliferation *in vitro*. Also, AKAP12 was co-regulated by MEG3/miR-29c axis. MEG3 mediated the aggressive behaviors of MEN cells via miR-29c/AKAP12 axis, supporting that MEG3 served as a promising biomarker for the diagnosis and treatment of human MEN.

## Introduction

Meningioma (MEN) belongs to the central nervous system disease with 30–40% morbidity (1). MEN is classified according to the classification of the World Health Organization (WHO). MEN is regarded as a type of general neoplasm that derived from the meningeal coverings of the brain or spinal cord. Although most of MEN is classified as benign tumors in qualitative classification (2, 3), the malignant MEN usually occurs with rapid growth and metastasis (4). Currently, continuous studies have achieved development on diagnosis and therapy for MEN, but the occurrence and recurrence of MEN is still pessimistic due to the ambiguous pathogenesis (5). Hence, it is important to discover the effective therapeutic strategies for MEN via understanding the pathogenesis and progression of MEN.

Among the transcriptome, the largest portion of gene is non-coding RNAs (ncRNAs). Also, long non-coding RNAs (lncRNAs) with over 200 nucleotides in length constitute the partial members of ncRNAs (6). Emerging evidence implied the extensive function of lncRNAs on the onset and development of diverse tumors, such as cell proliferation, metastasis, and recrudesce (7–9). Moreover, various lncRNAs were identified and confirmed to modify the pathological processes of MEN. Maternally expressed gene 3 (MEG3) is a well-known lncRNA, and it is commonly considered to be a tumor suppressor (10). For example, MEG3 impeded tumor growth of cervical carcinoma cells via promoting cell-cycle arrest and apoptosis (11). Furthermore, MEG3 was also associated with MEN, and it can constrain the tumorigenesis of MEN (12). Thus, we attracted more attention on the biological role of MEG3 in MEN progression.

Recently, microRNAs (miRNAs) are a type of post-transcriptional mediator that play the master roles in the modulation of gene expression (13). Moreover, miRNAs were demonstrated to be involved in cell-cycle arrest, apoptosis, and other physiological progressions (14). Multiple researches reported that miRNAs played key roles in healthy individuals and several cancers (15). Consequently, miRNAs are intensely considered as the biomarkers of diagnosis and prognosis in cancers (16). To be specific, miR-29c served as a tumor suppressor in nasopharyngeal carcinoma by targeting TIAM1 to suppress cell metastasis (17). Nevertheless, miR-29c-3p was validated to be upregulated in MEN tissues, implying that miR-29c restrained the development of MEN (18). In this study, we aimed to explore whether miR-29c could interact with MEG3 to regulate the pathogenesis and tumorigenesis of MEN. In addition, miRNAs exerted their functions via binding to the 3’-untranslated regions (3’UTRs) of targets (19). A-kinase anchor protein 12 (AKAP12) was confirmed to be closely implicated in MEN (20). In this paper, we determined the expression profiles of MEG3, miR-29c, and AKAP12 in MEN, and the work pathway among them was also expounded.

## Materials and Methods

### Clinical Specimens and Cell Culture

A total of 32 cases of human MEN tissues and 5 cases of normal meninges samples were collected from The First Affiliated Hospital of Fujian Medical University. The clinicopathologic features of MEN patients were presented in **Table 1**. All the available specimens were immediately stored at -80°C. Moreover, all written informed consents were gained from every participator before surgery, and this research was approved by the Ethics Committee of The First Affiliated Hospital of Fujian Medical University.

**Table 1:** Clinicopathological variables and MEG3 level in meningioma.

Clinicopathologic features	Relative MEG3 level		P value
	Low (%)	High (%)	
Age(years)			>0.05
≥55	10 (55.6)	8 (44.4)	
<55	8 (57.1)	6 (42.9)	
Gender			>0.05
Male	6 (50.0)	6 (50.0)	
Female	12 (60.0)	8 (40.0)	
Tumor side			>0.05
Right	13 (65.0)	7 (35.0)	
Left	5 (41.7)	7 (58.3)	
Tumor location			>0.05
Frontal and/or temporal lobes	14 (60.9)	9 (39.1)	
Other	4 (44.4)	5 (55.6)	

Malignant meningioma cell lines (IOMM-Lee and CH157-MN) were obtained from the Chinese Academy of Medical Sciences (Beijing, China). In this assay, the meningioma cells were maintained in Dulbecco’s Modified Eagle’s Medium (DMEM; Gibco, Carlsbad, CA, USA), supplemented with 10% fetal bovine serum (FBS; Gibco), and cultured in a humidiﬁed incubator containing 5% CO_2_ at 37°C.

### Vector and Oligonucleotide Transfection

Small interfering RNA (siRNA) targeting MEG3 (si-MEG3, 5’-GGAUGGCACUUGACCUAGA-3’), siRNA targeting AKAP12 (si-AKAP12, 5’-AGGUUAGUCACGCCAAGAA-3’), and the siRNA control (si-con) were purchased from Ribobio (Guangzhou, China). Also, the overexpression vector of MEG3 (MEG3) and its blank control (pcDNA) were obtained from Ribobio. All the oligonucleotides, including miR-29c mimic (miR-29c), miR-29c inhibitor (anti-miR-29c), and their relative controls (miR-con and anti-miR-con), were purchased from GenePharma (Shanghai, China). Transient transfection was carried out using Lipofectamine 3000 (Invitrogen, Carlsbad, CA, USA) as per the manuals.

### Quantitative Real-Time Polymerase Chain Reaction (qRT-PCR) Assay

Total RNA was harvested and extracted from clinical tissues and meningioma cell lines using Trizol reagent (Invitrogen) in accordance with the user’s guidebook. RNA was reversely transcribed into complementary DNA (cDNA) using PrimeScript RT Reagent Kit (TaKaRa, Dalian, China). Next, SYBR Premix Ex Taq (TaKaRa) was employed to perform qRT-PCR assay via mixture with equal cDNA, primers, and RNA-free water. Glyceraldehyde-3-phosphate dehydrogenase (GAPDH; for MEG3 and AKAP12) and U6 (for miR-29c) acted as the endogenous controls, and the relative level was assessed via the 2^-ΔΔCt^ method. The primers were provided by Sangon Biotech (Shanghai, China):

MEG3 (Forward: 5’-CTGCCCATCTACACCTCACG-3’, Reverse: 5’-CTCTCCGCCGTCTGCGCTAGGGGCT-3’); miR-29c (Forward: 5’-GCCTAGCACCATTTGAAATCG-3’, Reverse: 5’-GTGCAGGGTCCGAGGT-3’); AKAP12 (Forward: 5’-GGAATTCGATGGGCGCCGGGAGCTCCAC-3’, Reverse: 5’-CCGCTCGAGGTCATCTTCGTTGGCCCCTG-3’); GAPDH (Forward: 5’-ACTCCTCCACCTTTGACGC-3’, Reverse: 5’-GCTGTAGCCAAATTCGTTGTC-3’); U6 (Forward: 5’-CTCGCTTCGGCAGCACA-3’, Reverse: 5’-AACGCTTCACGAATTTGCGT-3’).

### Bioinformatics Analysis

LncBase V2.0 was used to predict the potential binding sites between MEG3 and miR-29c. StarBase showed that there were binding sites between miR-29c and AKAP12.

### Dual-Luciferase Reporter Assay

The sequences of MEG3 and 3’UTR of AKAP12 containing the binding sites of miR-29c were amplified and cloned into psiCHECK-2 vector (Promega, Madison, WI, USA), named as MEG3-WT and AKAP12-WT. The putative common fragments were replaced as indicated (MEG3-MUT and AKAP12-MUT) to mutant the predictive binding sites of MEG3 and AKAP12. IOMM-Lee and CH157-MN cells were seeded into a 24-well plate. Then, cells were co-transfected with the above-formed reporters and miR-29c or miR-con when cells reached ~70% confluence. The fluorescence intensities of the reporters were identified by the Dual-Luciferase Reporter Assay System (Promega) at 48 h post-transfection.

### RNA Immunoprecipitation (RIP) Assay

Magna RIP Kit (Millipore, Bedford, MA, USA) was used to analyze the relationship between miR-29c and MEG3 or AKAP12. First, IOMM-Lee and CH157-MN cells were lysed by the RIP lysis buffer, and then the lysate was incubated with the relative magnetic beads that conjugated with human anti-Argonaute2 (Ago2; Abcam, Cambridge, MA, USA) antibody or matched control antibody (IgG; Abcam). QRT-PCR was used to assess levels of MEG3, miR-29c, and AKAP12.

### Flow Cytometry Assay

MEN cells were re-suspended and fixed with 70% ethanol (ice-cold) for at least 1 h. Then, cells were re-suspended in HBSS (Hank’s balanced salt solution) supplemented with 50 µg/mL Propidium Iodide (PI; Sigma, St. Louis, MO, USA) and RNase A (Thermo Fisher Scientific, Rockford, IL, USA) and incubated for 1 h at room temperature without light. The ability of cell-cycle arrest was assessed using flow cytometry (FACS Calibur; BD Biosciences, San Jose, CA, USA).

### Wound Healing Assay

For wound healing assay, cells were plated into the 6-well plates, and a 200 µL pipette tip was employed to generate an artificial wound at 12 h post-inoculation. Then, the cells were incubated for 48 h, and the wound closure was observed and photographed. The migrated distance of the cell coverage across the initial wound was deemed to represent the migration rate.

### Transwell Assay

Cell invasion assay was performed using the transwell chamber (8 µm, Corning Costar, Corning, NY, USA). Briefly, the upper chamber was pro-coated with Matrigel (Corning Costar). Then, the transfected MEN cells (5×10^4^) were re-suspended with serum-free media and added into the upper transwell chamber. Meanwhile, to the lower chamber was added 600 µL complete medium. After incubation for 48 h, the non-invasion cells were erased, and the invaded cells were stained with 0.1% crystal violet (Sigma). The stain condition was photographed and quantitated via counting five random fields.

### Cell counting Kit-8 (CCK-8) Assay

The proliferation ability of MEN cells was assessed by CCK-8 assay. Seeded into each well were 5 × 10^3^ cells in 200 uL cell suspension of the 96-well plates. Then, cells were treated with 10 μL CCK-8 solution (Dojindo, Tokyo, Japan) and the absorbance was detected at 450 nm using Multiskan Go spectrophotometer (Thermo Fisher Scientific, Inc., Waltham, MA, USA).

### Western Blot Assay

As previously described (21), total proteins were isolated from MEN tissues and cells by RIPA lysis buffer (Beyotime, Shanghai, China). Next, sodium dodecyl sulfate-polyacrylamide gel (12%) was used to separate equal proteins, and the isolated proteins were transferred onto the nitrocellulose membrane (Millipore) and incubated with corresponding primary antibodies (Abcam): anti-AKAP12 (1:6000, ab9698) and β-actin (1:6000, ab8226). After incubation overnight at 4℃, the membrane was covered by the diluted secondary antibody for 40 min at room temperature. Then, the complex signals were visualized with enhanced chemiluminescence reagent (Millipore) and film exposure.

### Statistical Analysis

The data from the three independent assays were exhibited as mean ± standard deviation (SD). Difference comparison was analyzed using one-way analysis of variance (ANOVA; for three or more groups) or Student’s two-tailed *t*-test (for two-group), and Tukey test was selected as the post-hoc test for ANOVA. Pearson correlation analysis used to analyze the expression correlations among MEG3, miR-29c, and AKAP12. A *P*-value less than 0.05 was considered to be statistical significance.

## Results

### MEG3 Was Low-Expressed, While Mir-29c Was Upregulated in MEN Tissues

**Figure 1:**
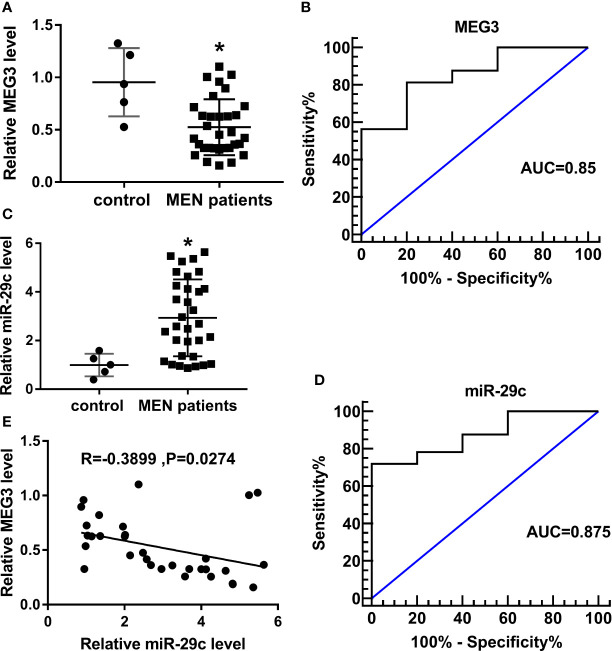
MEG3 was low-expressed, while miR-29c was upregulated in MEN tissues. **(A, C)** Relative levels of **(A)** MEG3 and **(C)** miR-29c in MEN tissues compared with normal control. **(B, D)** ROC curve about **(B)** MEG3 and **(D)** miR-29c in which MEN samples compared with matched control. **(E)** The correlation between miR-29c and MEG3 in MEN samples. *P<0.05.

The expression of MEG3 was determined in MEN specimens. Compared with normal control, a low level of MEG3 was observed in MEN tissues (**Figure 1A**). In addition, relative operating characteristic curves (ROC) analysis was carried out, and the area under the ROC curves (AUC; 0.85) showed that MEG3 might be a potential marker in MEN progression (**Figure 1B**). Moreover, miR-29c was increased in MEN tissues (**Figure 1C**). Similarly, the ROC curves implied the apparent isolation between MEN and match healthy donators, with an AUC of 0.875 for miR-29c (**Figure 1D**). From all subjects, we displayed that miR-29c was inversely correlated with MEG3 in clinical MEN tissues (**Figure 1E**). Collectively, MEG3 and miR-29c acted as strict factors in the process of MEN.

### MEG3 Was a Sponge of miR-29c

**Figure 2:**
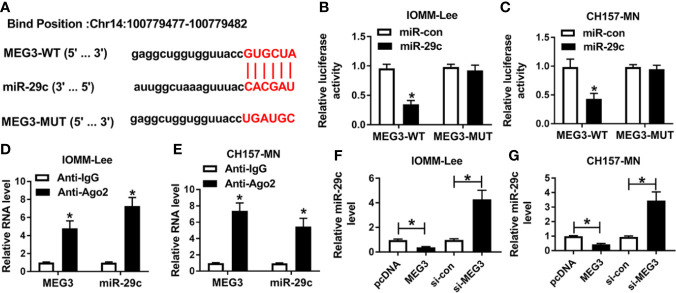
MEG3 was a sponge of miR-29c. **(A)** The predictive common fragments between MEG3 and miR-29c. **(B, C)** The luciferase activities of MEG3-WT and MEG3-MUT reporters in IOMM-Lee and CH157-MN cells with miR-29c or miR-con transfection. **(D, E)** RIP analysis for the relationship between miR-29c and MEG3. **(F, G)** Relative level of miR-29c in the two MEN cells under MEG3 or si-MEG3 introduction. *P<0.05.

According to the opposite expression between MEG3 and miR-29c, we speculated that MEG3 could regulate miR-29c. As described in **Figure 2A**, lncBase V2.0 predicted that there were the binding sites between MEG3 and miR-29c. Then, dual-luciferase reporter assay and RIP assay were performed to verify the interrelation between them. We found that the luciferase activity of MEG3-WT reporter was remarkably decreased (about 70%) in miR-29c-transfected IOMM-Lee and CH157-MN cells, whereas miR-29c had no statistical impact on the luciferase activity of mutant reporter (**Figures 2B, C**). As shown in **Figures 2D, E**, the levels of MEG3 and miR-29c were augmented in the Ago2-treated group. Next, MEG3 or si-MEG3 was transfected into IOMM-Lee and CH157-MN cells, respectively. QRT-PCR analysis exhibited that MEG3 passively regulated miR-29c in the two MEN cells (**Figures 2F, G**). All the results demonstrated that MEG3 served as the upstream of miR-29c in MEN cells.

### The Repressive Impact of MEG3 Increase on Cell-Cycle, Migration, Invasion, and Proliferation Was Overturned by miR-29c Upregulation in MEN Cells

**Figure 3:**
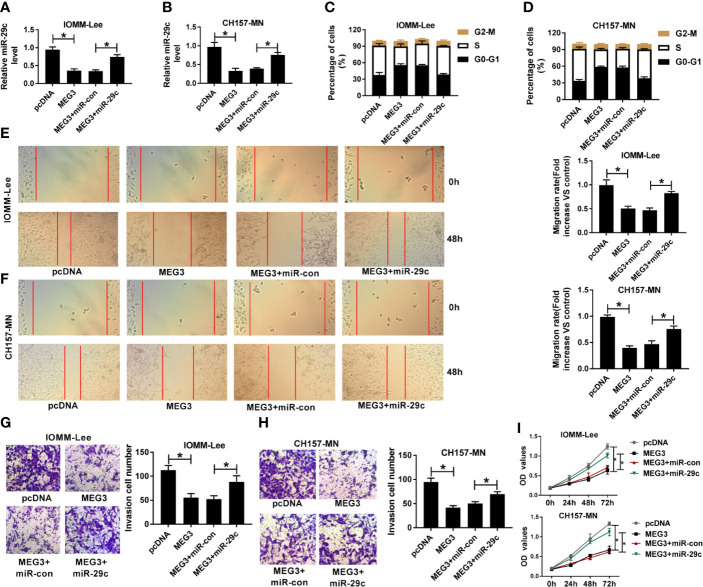
The repressive impact of MEG3 increase on cell-cycle, migration, invasion, and proliferation was overturned by miR-29c upregulation in MEN cells. **(A-I)** IOMM-Lee and CH157-MN cells were transfected with pcDNA, MEG3, MEG3+miR-con, or MEG3+miR-29c, respectively. **(A, B)** Relative level of miR-29c in MEN cells with MEG3 and miR-29c introduction. **(C, D)** Flow cytometry analysis for cell-cycle arrest in the two MEN cells. **(E, F)** Wound healing analysis for the influence of MEG3 or miR-29c increase on cell migration *in vitro*. **(G, H)** The capacity of cell invasion in IOMM-Lee and CH157-MN cells with MEG3 and miR-29c supplement. **(I)** Cell proliferation was detected by CCK-8 assay. *P<0.05.

Given the molecular mechanism between miR-29c and MEG3, we investigated the biological function of them in MEN cells. First, MEG3 alone or combined with miR-29c was transfected into IOMM-Lee and CH157-MN cells. As depicted in **Figures 3A, B**, MEG3 increase could impede the level of miR-29c in the two MEN cells, and reintroduction with miR-29c could overturn this effect. The triggered ability of cell-cycle arrest resulted from MEG3 augment was distinctly hindered via co-transfection with MEG and miR-29c in the two MEN cells (**Figures 3C, D**). Moreover, cell migration was assessed using wound healing assay, and the results determined that miR-29c supplement relieved the inhibitory effect of MEG3 on cell migration *in vitro* (**Figures 3E, F**). Also, cell invasion was restrained as a result of MEG3 increase, which was regained by miR-29c supplement in IOMM-Lee and CH157-MN cells (**Figures 3G, H**). MEG3 overexpression inhibited cell proliferation, which was reversed by miR-29c upregulation (**Figure 3I**). In brief, MEG3 regulated cell-cycle arrest, migration, invasion, and proliferation via miR-29c in MEN cells.

#### MiR-29c Directly Targeted AKAP12

**Figure 4:**
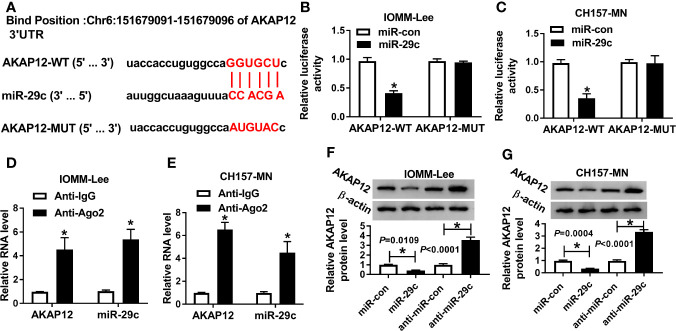
MiR-29c directly targeted AKAP12. **(A)** The binding sites between miR-29c and AKAP12. **(B, C)** Dual-luciferase reporter analysis for the interrelation between miR-29c and AKAP12. **(D, E)** Relative levels of miR-29c and AKAP12 in Ago2 or IgG-combined precipitates. **(F, G)** The role of miR-29c mimic or inhibitor in altering the protein level of mature AKAP12 in IOMM-Lee and CH157-MN cells. *P<0.05.

In view of the foregoing introduction, we attempted to seek the potential targets of miR-29c. After prediction with starBase software, we found that miR-29c possessed complementary sequence with AKAP12 (**Figure 4A**). Results from dual-luciferase reporter analysis showed that the luciferase activity of AKAP12-WT reporter was significantly diminished by miR-29c, but miR-29c had no statistical effect on changing luciferase activity of the mutant reporter system in IOMM-Lee and CH157-MN cells (**Figures 4B, C**). Levels of miR-29c and AKAP12 were notably upregulated in Ago2-treated IOMM-Lee and CH157-MN cells (**Figures 4D, E**). As shown in **Figures 4F, G**, miR-29c could inversely regulate the level of AKAP12 at the aspect of protein expression. Overall, we could conclude that AKAP12 acted as the downstream of miR-29c.

#### The Absence of AKAP12 Reversed The Reductive Effect Of Mir-29c Inhibitor n Cell-Cycle, Migration, Invasion, and Proliferation *In Vitro*

**Figure 5:**
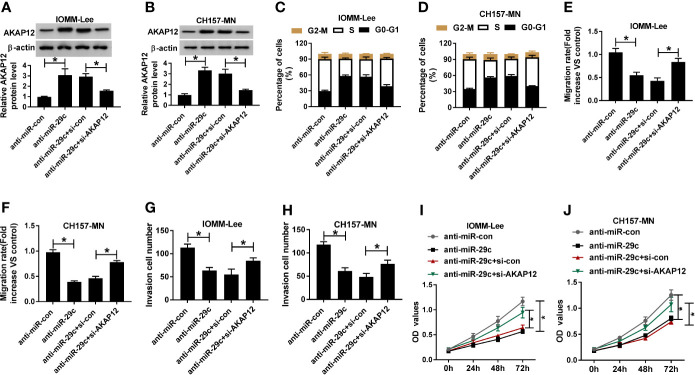
The absence of AKAP12 reversed the reductive effect of miR-29c inhibitor on cell-cycle, migration, invasion, and proliferation *in vitro*. **(A–J)** The anti-miR-con, anti-miR-29c, anti-miR-29c+si-con, or anti-miR-29c+si-AKAP12 was introduced into IOMM-Lee and CH157-MN cells, respectively. **(A, B)** Relative level of AKAP12 in selected MEN cells. **(C, D)** The effect of miR-29c or AKAP12 decrease on the alteration of cell-cycle arrest *in vitro*. **(E, F)** The ability of cell migration in anti-miR-29c or anti-miR-29c+si-AKAP12-transfected MEN cells. **(G, H)** Transwell analysis for the change of cell invasion in IOMM-Lee and CH157-MN cells with anti-miR-29c or anti-miR-29c+si-AKAP12 introduction. **(I, J)** Cell proliferation in MEN cells was determined by CCK-8 assay. *P<0.05.

Considering the molecular mechanism between miR-29c and AKAP12, we further researched the functional roles of them. First, anti-miR-29c alone or along with si-AKAP12 was introduced into IOMM-Lee and CH157-MN cells. AKAP12 silencing could abrogated miR-29c inhibitor-mediated promoting effect on the level of AKAP12 *in vitro* (**Figures 5A, B**). Then, functional assays were carried out, and flow cytometry analysis illustrated that cell-cycle arrest was reinforced as a result of miR-29c inhibition, and such promoting effect was abolished via simultaneous deficiency of AKAP12 in IOMM-Lee and CH157-MN cells (**Figures 5C, D**). Moreover, reintroduction with si-AKAP12 could eliminate the reductive impact of miR-29c inhibitor on cell migration and invasion in the two MEN cells (**Figures 5E–H**). Furthermore, AKAP12 knockdown could abolish the inhibition effect of miR-29c inhibitor on cell proliferation (**Figures 5I, J**). Namely, miR-29c modified cell behaviors, including cell-cycle arrest, migration, invasion, and proliferation via targeting AKAP12 in MEN progression.

### AKAP12 Was Co-Regulated by MEG3 and miR-29c

**Figure 6:**
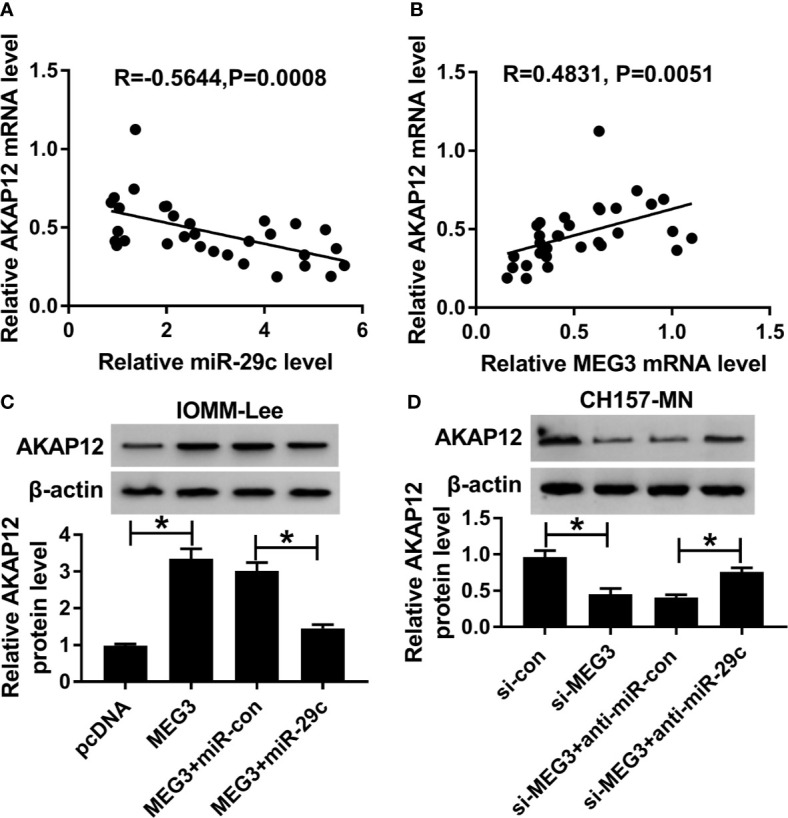
AKAP12 was co-regulated by MEG3 and miR-29c. **(A, B)** The correlation between AKAP12 and **(A)** miR-29c or **(B)** MEG3 in clinical MEN tissues. **(C, D)** PcDNA, MEG3, MEG3+miR-con, or MEG3+miR-29c was transfected into IOMM-Lee and CH157-MN cells, respectively. Relative level of AKAP12 in treated MEN cells. *P<0.05.

As mentioned above, we were devoted to exploring the regulatory mechanism systematically. As described in **Figure 6A**, an inverse correlation between miR-29c and AKAP12 was observed in clinical MEN tissues. On the contrary, AKAP12 was positively associated with MEG3 in MEN samples (**Figure 6B**). Then, MEG3 alone or combined with miR-29c was transfected into IOMM-Lee and CH157-MN cells, and the high level of AKAP12, caused by MEG3 increase, was apparently decreased after co-transfection with miR-29c and MEG3 in the two MEN cells (**Figure 6C**). Similarly, the declined level of AKAP12 induced by si-MEG3 was restored by anti-miR-29c (**Figure 6D**). Collectively, AKAP12 was co-regulated by MEG3 and miR-29c in the process of MEN.

## Discussion

The interaction between lncRNAs and human cancers has been generally illustrated by increasing researches, suggesting the critical functions on epigenetic modulation of human phenotypes (8, 9, 22). MEN, especially malignant MEN (high-grade), is characterized by migrated and invasive capacity. Therefore, the underlying role of lncRNAs in MEN process is complex and ambiguous, but attractive. And there are still other possible transformations of MEN from benign to malignancy.

As previously described, lncRNAs were believed to participate in multiple human diseases, including cardiovascular, endocrine system disease, and tumors (23, 24). For example, LINC00341 reinforced cell proliferation and migration of vascular smooth muscle cells by targeting miR-214 (25). The tumor growth and metastasis of MEN were modulated by the LINC00460/miR-539 axis *in vitro* (26). MEN is a type of brain disease with a large proportion as benign. However, the occurrence of the rapid invasion capacity becomes a serious barrier for human health. In consideration of the critical function of lncRNAs in tumorigenesis and pathogenesis, we attempted to discover the influence of unique lncRNA in the progression and initiation of MEN. In the present research, we determined that MEG3 was expressed at a low level in clinical MEN specimens and cell lines (IOMM-Lee and CH157-MN) with respect to the matched controls, indicating the possible tumor-suppressive role of MEG3 in aggressive phenotypes. A previous report manifested that MEG3 could retard aggressive behaviors, including cell proliferation, migration, and invasion by sponging miR-19a in glioma cells (22). Currently, the supplement of MEG3 acted as a tumor suppressor, showing as the blockage of cell-cycle, migration, invasion, and proliferation in MEN cells. Moreover, the incidence of cell migration *in vivo* is related to mostly Grade II (atypical) and Grade III (malignant) meningioma (27). Our results provided that MEG3 could regulate cell migration in MEN cells, indicating a potential biomarker for the treatment of MEN.

Until now, the well-known pattern of lncRNAs mediating the carcinogenesis is the competing endogenous RNA (ceRNA) of miRNAs. Especially, lncRNAs served as the sponge of miRNAs to separate the abundance of target miRNAs (28–30). According to the above description, we tried to expose the partial work pathway of MEG3 in MEN. We found that miR-29c was a potential target of MEG3. In the present investigation, an evident high expression of miR-29c was observed in MEN tissues and cell lines in comparison with matched controls. Previous research implied that miR-29c-3p regulated the pathogenesis of MEN by mediating pentraxin 3 (PTX3) (18). In addition, miR-29c acted as cancer-associated miRNA and could modulate the tumorigenesis of multiple human carcinomas. For instance, miR-29c deletion was tightly implicated in poor prognosis in laryngeal squamous cell carcinoma (31). Also, another report presented that miR-29c retarded cell migration and invasion by inactivating cyclin-dependent kinase 6 (CDK6) in gastric cancer (32). Currently, the interrelation between MEG3 and miR-29c was illustrated by means of the dual-luciferase reporter and RIP assays. Also, an inverse correlation between miR-29c and MEG3 was determined. Subsequently, functional assays were conducted to explore the biological role of miR-29c in affecting the process of MEN *in vitro*. Furthermore, the reductive impact of MEG3 increase on cell-cycle, migration, invasion, and proliferation was eliminated after co-transfection with miR-29c mimic in IOMM-Lee and CH157-MN cells. Moreover, ROC analysis indicated that MEG and miR-29c might be the biomarkers for the diagnosis of MEN.

Accruing findings have disclosed that miRNAs exerted their function by repressing the expression or transcription of special mRNAs (33). Consequently, finding the underlying targets might serve as a novel insight for genetic therapy. As predicted by starBase software, AKAP12 possessed some binding sites of miR-29c. AKAP12 was regarded to be strictly connected with several human cancers (34). A previous research expounded that the absence of AKAP12 caused the augment of cell proliferation and metastasis and conferred an anaplastic profile in MEN cells (20). Consistently, we agreed that AKAP12 suppressed the aggressive phenotypes of MEN cells. In the current study, we proved that miR-29c negatively regulated AKAP12 expression. Indeed, the inhibition of miR-29c declined cell-cycle and mobility of MEN cells, and such repressive influence of anti-miR-29c could be abolished via co-transfection with si-AKAP12 *in vitro*. Apart from that, AKAP12 was co-modulated by miR-29c and MEG3 in the two MEN cells.

In general, this present study revealed that MEG3 was down-regulated in MEN tissues and cells, serving as a tumor-suppressive lncRNA in MEN malignancy. MEG3 declined the expression of AKAP12 by targeting miR-29c to block cell-cycle, migration, invasion, and proliferation *in vitro* and might supply a novel biomarker for the treatment of MEN.

## Data Availability Statement

The datasets generated for this study are available on request to the corresponding authors.

## Ethics Statement

The studies involving human participants were reviewed and approved by This study was approved by The First Affiliated Hospital of Fujian Medical University Ethics Committee. Informed consents were obtained from the parents or their guardians in accordance with the Declaration of Helsinki. The patients/participants provided their written informed consent to participate in this study.

## Author Contributions

CD, XY, and JX performed and designed experiments, analyzed, and interpreted data, and wrote the manuscript. DK, ZH, XB, DW, HG, and GZ performed the experiments. JG, DK, and XW contributed to discussions and reviewed and edited the manuscript. All authors contributed to the article and approved the submitted version.

## Funding

XW: Special Health Fund of Fujian Provincial Department of Finance (BPB-20100201-1, BPB-WXY2011, BPB-WXY2014, BPB-WXY2014-2) and Professor Fund of Fujian Medical University (JS15013). CD: National Natural Science Foundation of China (No. 81901395).

## Conflict of Interest

The authors declare that the research was conducted in the absence of any commercial or financial relationships that could be construed as a potential conflict of interest.
